# Supplementary material for: Cellular anatomy of the mouse primary motor cortex
Source: Nature. 2021 Oct 6;598(7879):159–66. doi: 10.1038/s41586-021-03970-w (PMC8494646; doi:10.1038/s41586-021-03970-w)
Supplement: Supplementary file 1 — This file contains further details on methods and results regarding (1) automated MOp-SSp boundary detection on Nissl stained brain sections (P.P.M. laboratory at CSHL); (2) eFLASH-based 3D immunohistochemistry (K.C. laboratory at MIT); (3) integration of labels from existing atlases onto the Allen CCF (Y.K. laboratory at Penn State University); (4) supplementary information for tract tracing and viral labelling experiments (H.-W.D. laboratory at UCLA); (5) Supplementary Methods and data analysis for BARseq (including Supplementary Tables 6–8) (A.M.Z. laboratory at CSHL). [file 41586_2021_3970_MOESM1_ESM.pdf]

nature portfolio

https://doi.org/10.1038/s41586-021-03970-w

Supplementary information

Cellular anatomy of the mouse primary motor cortex

In the format provided by the authors and unedited

SUPPLEMENTARY INFORMATION

Automated MOp-SSp boundary detection on Nissl stained brain sections (Partha Mitra Lab at CSHL)

Animal subjects

Ten wildtype male C57BL/6J mice (11-20 weeks, weight 20-26.4g) were used for cytoarchitectural analysis of the MOp-SSp border variation.

Histology and immunohistochemical processing

The entire brain was sectioned coronally with 20 µm thickness using the tape-transfer method to minimize tissue distortion. In one brain, consecutive coronal sections were processed with Nissl staining for cytoarchitecture. In all other brains, alternative coronal sections were processed with Nissl staining.

Imaging and post-acquisition processing

Bright field imaging was performed using Nanozoomer 2.0 HT (Hamamatsu, Japan) with a 20x objective. Images of individual coronal sections were converted into 8-bit RGB images at 0.46 µm/pixel in-plane resolution in JPEG2000 format (**Figure S1A**).

Data Analysis

A diffeomorphic registration pipeline[1] was applied to register the whole stack of coronal sections into a 3D volume and mapped with the Allen Mouse Brain Atlas[2]. Two versions of atlas annotation were applied: Common Coordinate Framework (CCF) v2 published in 2011[3] and CCFv3 published in 2017[4]. For sections within a range of AP -1.5mm ~ +1.5mm (**Figure S1A**), a thresholding method was used to eliminate the high-intensity pixels in the red and green channels and retain the full spectrum blue channel intensities. The segmented Nissl-stained cells in the whole section constitute a binary mask M_N (**Figure S1B**). The binary cortical mask, R_{ctx}, was extracted from the mapped CCFv3 segmentation. A morphological thinning operation was applied on the boundary-smoothed R_{ctx} to generate a smooth pseudo medial axis ($medAX_s$) of the entire cortical band. A centroid C_{ctx} was determined based on R_{ctx}. The angle ϕ_s^{medAx} to each point sampled uniformly on $medAX_s$ was calculated clockwise from the C_{ctx} to generate the parameterized representation of $medAX_s$ (**Figure S1C**).

$$\phi_s^{medAx}(i) = \tan^{-1}\frac{\|medAx_s(y_i) - C_{ctx}(y)\|}{\|medAx_s(x_i) - C_{ctx}(x)\|} \qquad \textit{Eq.1}$$

The cortical normal N_{medAx} at each point on the $medAX_s$ was sampled with a uniformly distributed 1,000 points spanning the cortical depth, to determine the profile representation of the flattened cortex map. For a point $N_{medAx} \coloneqq \{x_i, y_i\}$ on the medial axis, the equation for the points on the image that fall on the normal is given by the equation

$$y = -\frac{medAx_s(x_{i+1}) - medAx_s(x_i)}{medAx_s(y_{i+1}) - medAx_s(y_i)}(x - medAx_s(x_i)) + medAx_s(y_i) \qquad \textit{Eq.2}$$

Figure S1D shows an example of cortical normals N_{medAx} sampled at 1,000 cortical depths of the Nissl mask M_N for all angles ϕ_s^{medAx}. The intensity at a given point in this image is the density of segmented Nissl mask estimated with a neighborhood of ~93x93µm[2].

To identify the MOp border with cell-sparse SSp layer 5a below a densely-packed granular cell layer 4, we looked at the fractional change of the flattened cortical profile across cortical depth

$$dN(D_i, \phi_{s,i}^{medAx}) = \frac{N\left(D_i, \phi_{s,i}^{medAx}\right) - N(D_{i-1}, \phi_{s,i}^{medAx})}{N(D_i, \phi_{s,i}^{medAx})}. \qquad \textit{Eq.3}$$

All local minima were identified within the middle 30%-70% of cortical depth in dN. Putative layer 5a of SSP were determined by connecting the minima of these cell density profiles (**Figure S1E**). By aligning all profiles in the stack ranging from -1.5mm to +1.5mm from Bregma in AP direction, two smooth objects of putative SSp layer 5a were constructed in 3D (**Figure S1F**). The border B_n with MOp was identified as N_{medAx} at

ϕ_s^{medAx} associated with the boundaries of these objects on each profile (red lines in **Figure S1F**). B_n of all flattened cortical profiles were projected back to the registered brain space (curved cortex) and constructed a smooth surface $\boldsymbol{S_B}$ (**Figure S1G**). By reverse transformation of atlas mapping, $\boldsymbol{S_B}$ was projected into the common atlas space. The intersecting line between $\boldsymbol{S_B}$ and the dorsal surface of the cortex was compared with that of CCFv2 and CCFv3 in the subsequent analysis (**Extended Data Fig. 2c,d**).

Data Presentation

Cytoarchitecture image data are published online as part of the Mouse Brain Architecture web portal (http://brainarchitecture.org/). Links to individual datasets are available at https://docs.google.com/spreadsheets/d/1MPJuzSD-vGTJyuaj5SBUx345_K7MShwWy0zInPAbwSc/edit?usp=sharing.

Informatics Tools and Code availability

All related code for border detection are published on Github (https://github.com/bingxinghuo/AutoSegmentBrain/).

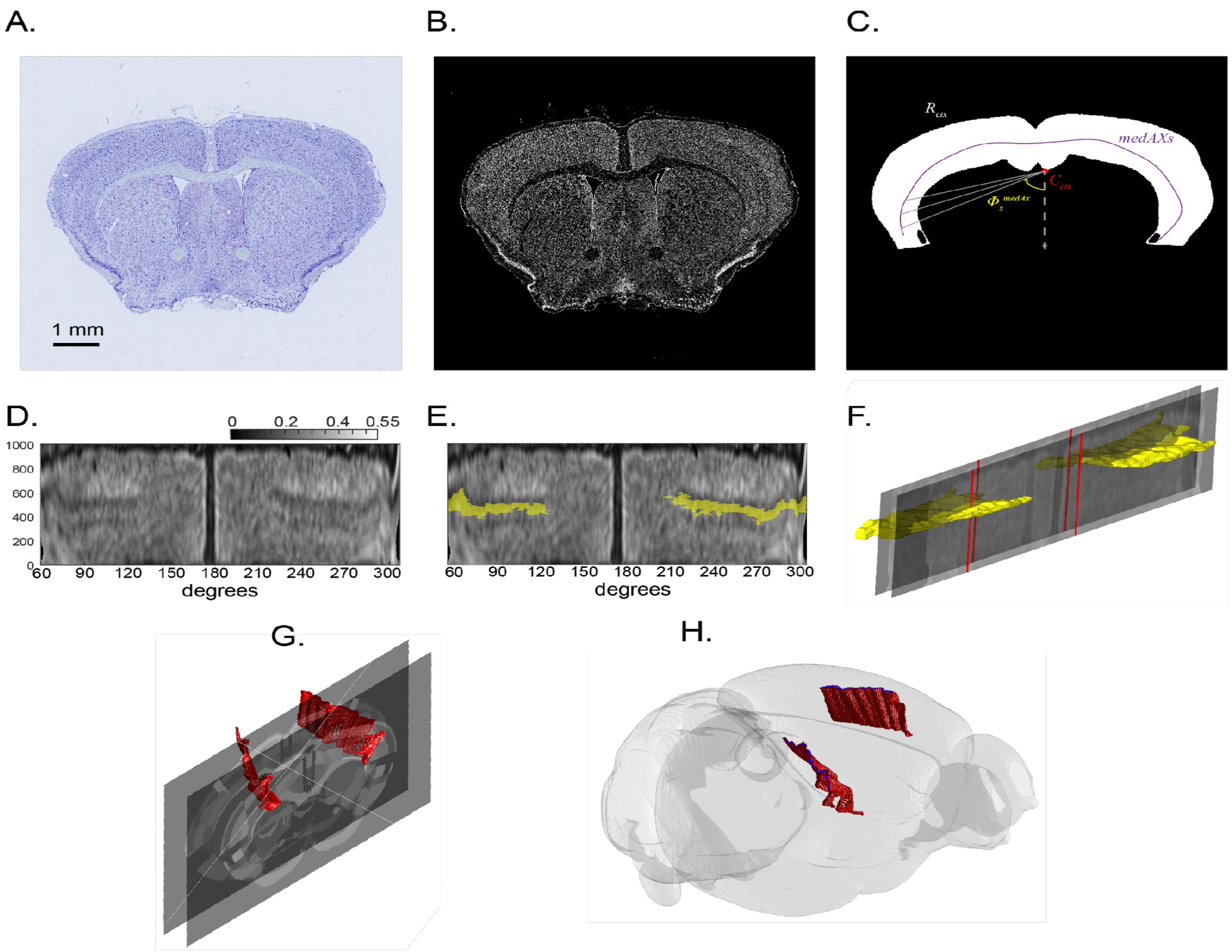

Supplementary Figure 1. A, Nissl-stained coronal section ~ +0.3 mm anterior to bregma. **B,** A cell segmentation mask obtained from the Nissl-stained section in **A** using color thresholding. **C,** Cortical mask based on registered CCFv3 segmentation. Medial axis $medAX_s$ (purple) and centroid C_{ctx} (red) were calculated based on the cortical mask. The phase shift of each sample point on $medAX_s$ from C_{ctx} was represented as angle ϕ_s^{medAx}. **D,** The cortical profile where the cortex was represented with angle ϕ_s^{medAx} in x-axis and cortical normal N_{medAx} in y-axis. The intensity of each point reflects the average cell density within the (ϕ_s^{medAx}, N_{medAx}) window.

E, Putative SSp layer 5a (yellow) detected in the flattened cortical profile. **F**, 3D representation of SSp layer 5a detected for all sections. Red lines show the boundary N_{medAx} associated with the middle bounds on either 3D object. **G**, 3D representation of the detected surface smoothened in registered brain space (red). Individual brain sections were mapped with atlas segmentation. **H**, 3D representation of the boundary surface (red) in atlas space. The intersection with the cortical surface is represented with blue lines.

eFLASH-based 3D immunohistochemistry (Kwanghun Chung lab at MIT)

Animal subjects

Young adult male mice (C57BL/6J) were purchased from Jackson (Stock No. 000664) and were housed in a 12 hr light/dark cycle with unrestricted access to food and water. All experimental protocols were approved by the MIT Institutional Animal Care and Use Committee and the Division of Comparative Medicine and were in accordance with the guidelines from the National Institute of Health.

Histology and immunohistochemical processing

Mouse brains were preserved using SHIELD technology[5]. Briefly, mice were transcardially perfused at 57 days of age with ice-cold 1X PBS and then with SHIELD perfusion solution (10% (w/v) P3PE and 4% PFA (w/v) in 1X PBS). The brains were dissected and incubated in the SHIELD perfusion solution at 4 °C for 48 h, and then transferred to the SHIELD-OFF solution (1X PBS containing 10% (w/v) P3PE). After incubation at 4 °C for 24 h, the brains were placed in the SHIELD-ON solution (0.1 M sodium carbonate buffer at pH 10) prewarmed to 37 °C and then incubated at 37 °C for 24 h. The brains were then washed in 1X PBS with 0.02% sodium azide at room temperature overnight and were rapidly cleared using stochastic electrotransport (SmartClear Pro, LifeCanvas Technologies).

SHIELD-processed brains were immunostained using eFLASH[6]. A Neurofilament-M antibody (MCA-3H11, Encor Biotechnology) and NeuN antibody (266004, Synaptic Systems) were used to immunostain a whole mouse brain. After staining, brains were washed in 1X PBS with 0.02% (w/v) sodium azide at RT overnight, and were fixed with 4% (w/v) PFA solution in 1X PBS at RT overnight to prevent the dissociation of bound antibodies. After washing with 1X PBS with 0.02% (w/v) sodium azide at RT with multiple solution exchanges, brains were optically cleared.

Protos-based immersion medium was used for optical clearing of whole brain[5]. Brains were firstly incubated in half-step solution (50/50 mix of 2X PBS and the Protos-based immersion medium) at 37°C overnight. Afterwards, the samples were moved to the pure immersion medium and incubated at 37°C overnight.

Imaging and post-acquisition processing.

Optically cleared brains were imaged with an axially-swept light-sheet microscope (SmartSPIM, Lifecanvas Technologies). Imaging was done using 3.6x objective (custom Lifecanvas design; 0.2NA, 12mm working distance, 1.8um lateral resolution) and using three lasers (488um, 561um, and 642um wavelengths). Acquired data was post-processed and region-segmented as described in Swaney et al[7]. Acquired 3D images were aligned to the CCFv3 based on tissue autofluorescence from 488nm laser illumination. Delineation of cortical layers in MOp was refined using anti-NeuN and anti-Neurofilament-M signals (**Extended Data Figure 2e**). Calculations and visualizations were done using Imaris (Bitplane) and Nuggt python package[7].

Integration of labels from existing atlases onto the Allen CCF (Yongsoo Kim lab at Penn State University)

Initially we used previously established ARA labels on a 3D reference brain based on serial two-photon tomography (STPT) imaging[8]. However, because images for our previous reference brain and the latest Allen Common Coordinate Framework (CCFv3) were acquired with STPT imaging, image registration (Elastix) of the Allen Reference Atlas[9] onto the CCF was seamless. We then manually drew segmentation lines using a vector drawing tool (Adobe Illustrator) based on the registered ARA. Although the registered ARA produced a rough estimate of ARA based segmentation in the CCF, fine adjustment by experts was required to ensure quality of the labels. Therefore we carefully reviewed each segmentation line to obtain precise ARA

segmentations on the CCF, as we have done previously for Franklin-Paxinos labels imported into the CCF[10] (**Extended Data Fig. 2f**). Drawings were overlaid on full resolution CCF images (10μm, x and y) in every 100 μm z interval. Fine adjustment of segmentation lines was performed based on autofluorescence contrast in the CCF and more than 10 different cell type-specific transgenic marker brains[10]. The complete set of CCF imaging templates with ARA delineations are available at https://kimlab.io/data_share/files/ARA_on_CCF/2021_ARA_on_CCF_forPublication.7z

Supplementary Information for Tract Tracing and Viral Labeling Experiments

Stereotaxic surgeries (Dong lab)

All Mouse Connectome Project (MCP) tract-tracing experiments were performed using 8-week old male C57BL/6J mice (Jackson Laboratories). For anterograde trans-synaptic tracing experiments and AAVretro injections in spinal cord, Ai14 tdTomato Cre-reporter mice were used (Jackson Laboratories, stock #007914, aged 2-3 months old).

On the day of the experiment, mice were deeply anesthetized and mounted into a Kopf stereotaxic apparatus where they were maintained under isofluorane gas anesthesia (Datex-Ohmeda vaporizer). For triple anterograde injection experiments, PHAL was iontophoretically delivered via glass micropipettes (inner tip diameter 24-32μm) using alternating 7 sec on/off pulsed positive electrical current (Stoelting Co. current source) for 10min, and AAVs were delivered via the same method for 2 min (inner tip diameter 8-12μm). For anterograde/retrograde co-injection experiments, tracer cocktails were iontophoretically delivered via glass micropipettes (inner tip diameter 28-32μm) using alternating 7 sec on/off pulsed positive electrical current (Stoelting Co. current source) for 10 min (PHAL/CTB-647). For quadruple retrograde tracing experiments, 50 nl of retrograde tracers were individually pressure-injected via glass micropipettes at a rate of 10nl/min (Drummond Nanoject III). Pressure injections were also similarly performed for anterograde transsynaptic experiments using scAAV1-hSyn-Cre (100 nl), labeling of presynaptic boutons with AAV-hSyn-mRuby2-sypEGFP (50 nL), and spinal injections of AAVretro-hSyn-GFP-WPRE and AAVretro-hSyn-Cre-WPRE (cervical and lumbar, respectively, 200 nL each; see Zingg et al., 2020 for detailed procedure). For TRIO experiments, AAVretro-hSyn-Cre-WPRE was injected into one MOp-ul downstream projection target (i.e., caudoputamen). And, Cre-dependent, TVA- and RG-expressing helper virus (AAV8-hSyn-FLEX-TVA-P2A-GFP-2A-oG) was injected into the MOp-ul. Animals were then survival for 2 weeks before receiving injections of mCherry-expressing G-deleted rabies virus in the MOp-ul. All injections were placed in the right hemisphere. Injection site coordinates for each surgery case are on the MCP iConnectome viewer (www.MouseConnectome.org).

Histology and immunohistochemical processing (Dong Lab)

After 1-3 weeks post-surgery, each mouse was deeply anesthetized with an overdose of Euthasol (pentobarbital) and trans-cardially perfused with 50ml of 0.9% saline solution followed by 50ml of 4% paraformaldehyde (PFA, pH 9.5). Following extraction, brain tissue was post-fixed in 4% PFA for 24-48hr at 4°C. Fixed brains were embedded in 3% Type I-B agarose (Sigma-Aldrich) and sliced into four series of 50μm thick coronal sections using a Compresstome (VF-700, Precisionary Instruments, Greenville, NC) and stored in cryopreservant at -20°C. For double co-injection experiments, one series of tissue sections was processed for immunofluorescent tracer localization. For PHAL or AAVretro-EF1a-Cre immunostaining, sections were placed in a blocking solution containing normal donkey serum (Vector Laboratories) and Triton X-100 (VWR) for 1 hr. After rinsing in buffer, sections were incubated in PHAL primary antiserum (1:1000 rabbit anti-PHAL antibody (Vector Laboratories Cat# AS-2300, RRID:AB_2313686)) or AAVretro-EF1a-Cre primary antiserum (1:1000 mouse-anti-Cre) mixed with blocking solution for 48-72 hours at 4°C. Sections were then rinsed again in buffer solution and then immersed in secondary antibody solution (blocking solution and 1:500 donkey anti-mouse IgG conjugated with Alexa Fluor 488 (Thermo Fisher Scientific Cat# A-21202, RRID:AB_141607) , or 1:500 donkey anti-rabbit conjugated with CY3 (Jackson ImmunoResearch Labs Cat# 715-165-151, RRID:AB_2315777) for 3 hrs. Finally, all sections were stained with Neurotrace 435/455 (Thermo Fisher Cat#

N21479) for 2-3 hours to visualize cytoarchitecture. After that, sections were mounted onto glass slides and cover slipped using 65% glycerol for data collection and analysis as described in **Methods**.

Regional output from MOp-ul mapped using PHAL(Dong Lab)

The following description of MOp-ul outputs is based primarily on experiment SW191201-01A. This experiment was selected as the most representative because the PHAL injection site had the most coverage of the MOp-ul region and was also the most restricted to it. The injection site was centered at the following coordinates: 0.5 mm anterior to bregma, 1.5 mm lateral, and 0.5 mm ventral (see **Fig. 1b**, right inset and **Extended Data Fig. 5c**). Three additional experiments, with substantial MOp-ul inclusion and restriction, are available for viewing at www.MouseConnectome.org (SW110516-01A, SW110321-01B, and SW110210-03A). MOp-ul projection pathways and their major terminals are described below in four subsections corresponding to four major rostral-caudal and topographic divisions of the central nervous system: (1) cerebral cortex, (2) cerebral nuclei (or basal ganglia), (3) interbrain (hypothalamus and thalamus), and (4) midbrain (tegmentum and tectum) and rhombic brain (including the cerebellum, pons, medulla, and spinal cord). MOp-ul sends output connections to > 110 gray matter regions, mostly bilaterally, but with predominant ipsilateral outputs to the injected side, along a pathway that extends to the spinal cord.

(1) Cerebral cortex. MOp-ul outputs follow three main routes to innervate other regions of the cerebral cortex: rostral, lateral, and caudal. First, the robust laterally-projecting MOp-ul pathway innervates the upper limb field of the laterally adjacent primary somatosensory area (SSp-ul) (**Extended Data Fig. 5d**). Second, the rostrally-projecting MOp-ul pathway targets the secondary somatomotor area (MOs, **Extended Data Fig. 5a,b**), with densest innervation near the frontal pole in a region that corresponds approximately to a previously delineated rostral forelimb area[11]. Some of the rostrally-projecting MOp-ul axons turn ventrally to innervate the claustrum (CLA), and more rostrally they reach the orbital region, there innervating mostly the lateral orbital area (ORBl) (**Extended Data Fig. 5a,b**). Third, the caudally-projecting MOp-ul pathway (via layer 6) generates a multilayer columnar terminal field in the SSp barrel field (SSp-bfd), and further laterally in the supplemental somatosensory area (SSs) (**Extended Data Fig. 5e**). In addition, MOp-ul axons extending laterally and ventrally along the caudal path provide substantial input to four additional cerebral cortical regions: perirhinal area (PERI), ectorhinal area (ECT), temporal association areas (TEa), and ventral auditory areas (AUDv).

Several other regions of the cerebral cortex receive light input from MOp including (in the cortical plate) the postsubiculum (POST), lateral entorhinal area (ENTl), retrosplenial area (RSP), posterior parietal association areas (PTLp), and (in the cortical subplate) the basolateral amygdalar nucleus anterior part (BLAa). Also noteworthy is a light intrinsic MOp connection from the MOp-ul to MOp subregions associated with motor control of the trunk (MOp-tr), lower limbs (MOp-ll), mouth (MOp-m) and nose (MOp-n). The intra-cerebral cortex connections of the MOp-ul are bilateral. Associational (ipsilateral) connections generally predominate, but commissural (contralateral) connections via the corpus callosum (mainly between 0.5 mm rostral and caudal to bregma) are also substantial, and, generally mirror the pattern of associational connections. Notable exceptions to this are commissural MOp-ul connections to SSp-bfd that are substantially weaker than the associational connection, and commissural MOp-ul connections to PERI, ECT, TEa and CLA that are noticeably stronger. To the best of our knowledge, these contralaterally predominant asymmetric projections have not been reported previously.

(2) Cerebral nuclei (basal ganglia). The MOp-ul provides substantial and broad input to the cerebral nuclei (mostly bilateral, but with exceptions noted below), including to both of its two major subdivisions, striatum and pallidum (**Extended Data Fig. 5c,d**). The CP (the dorsal striatum in rodents) receives strong MOp-ul projections. Notably, the present results, with our focus on MOp-ul connections, are consistent with our earlier report of MOp-ul connections to the lateral CP[12]. An additional striatal MOp-ul target is the nucleus accumbens (ACB), reached by MOp-ul axons that enter the ACB via the lateral edge of the CP to innervate the ACB shell region as well as a lateral part of the ACB core region. At a caudal CP level, a moderate MOp-ul pathway via the CP ventral tip reaches the central amygdalar nucleus where it forms a cluster of terminals in the capsular part (CEAc). MOp-ul inputs to pallidum regions include a relatively thin pathway that generates a dense terminal field in the medial segment of the globus pallidus (GPe), and a circumscribed bilateral input via the temporal limb of the anterior commissure to the bed nucleus of anterior commissure (BAC) (another connection that does not appear to have been previously reported).

(3) Interbrain. The thalamus is a major recipient of MOp-ul input via the internal capsule. Dense terminal fields are present in a rostrodorsal region of the reticular thalamic nucleus (RT), and the pathway extends to densely innervate the ventral anterior-lateral thalamic complex (VAL) (**Figure 1b, right; Extended Data Fig. 5e**). These results are consistent with a recent study in mice of the corticothalamic connections of layer 6b neurons in several cerebral cortical regions, including somatomotor areas [13]. They are also consistent with earlier studies in rats and other mammals [14,15].

Further caudal in the thalamus, the MOp-ul pathway to the RT and VAL extends ventrally to innervate the ventromedial thalamic nucleus (VM), and dorsally to densely innervate the posterior thalamic complex (PO), the paracentral- (PCN) and central lateral- (CL) thalamic nuclei, the mediodorsal thalamic nucleus lateral part (MDl), and, more caudally, the parafascicular nucleus (PF). Each of these MOp-ul connections is characterized by subregional topography that is distinct from the subregional topography of thalamic inputs arising from the SSp and MOs (**Figure 1b, right; Extended Data Fig. 5e,f**). The MOp-ul also gives rise to a cluster of terminals in the dorsal part of the ventral posteromedial thalamic nucleus (VPM), and a sparse innervation of the ventral posterolateral thalamic nucleus (VPL, that receives dense inputs from the SSp) (**Figure 1b, right; Extended Data Fig. 5e**). In addition to these robust MOp-ul connections, the MOp-ul also lightly innervates the nucleus reuniens (RE), rhomboid nucleus (RH), central medial thalamic nucleus (CM), ventral posteromedial thalamic nucleus parvicellular part (VPMpc), and subparafascicular nucleus parvicellular part (SPFp).

Also within the interbrain, a single hypothalamic region, the subthalamic nucleus (STN), receives MOp-ul input via the cerebral peduncle that is restricted to a narrow band of the STN (**Extended Data Fig. 5f**). Extending from the STN, some MOp-ul axons then innervate densely a ventromedial region of the adjacent zona incerta (ZI) (**Extended Data Fig. 5f**) before turning medially and dorsally and continuing caudally into the midbrain.

(4) Midbrain and lower brainstem. MOp-ul axonal projection entering the midbrain from the thalamus provides substantial input to several regions of the tegmentum, including the red nucleus (RN), midbrain reticular nucleus (MRN), nucleus of the optic tract (NOT), nucleus of the posterior commissure (NPC), and anterior pretectal nucleus (APN) (**Extended Data Fig. 5g**). Additionally, MOp-ul axons extending dorsally and medially from the MRN innervate a rostromedial division of the periaqueductal gray (PAGrm) immediately ventral to the nucleus of Darkschewitsch and provide extensive input to the PAG ventrolateral column (PAGvl, **Extended Data Fig. 5h**). The tectum also receives an input from MOp-ul, as a terminal continuation of the tegmental pathway, that targets a lateral part of the superior colliculus (SC) (**Extended Data Fig. 5h**).

The remaining MOp-ul axonal pathway traveling through the cerebral peduncle continues caudally to enter the hindbrain, where it provides input to several pontine regions, including dense terminal clusters in the pontine gray (PG) (**Extended Data Fig. 5h**) and lighter and more diffuse inputs to the tegmental reticular nucleus (TRN), pontine reticular nucleus (PRN), pedunculopontine nucleus (PPN), principal nucleus of the trigeminal nerve (PSV), and nucleus y (y). Furthermore, an MOp-ul connection reaches the pontine central gray (PCG) as a caudal continuation of MOp-ul axons that innervate the PAG, and a very sparse projection extends dorsally into the cerebellar nuclei.

Continuing caudally from the pons, a substantial MOp-ul projection enters and innervates the medulla (bilaterally for the most part), principally via branches of a main pathway projecting caudally along the pyramidal tract (**Extended Data Fig. 5i,j,k**). Moderate to dense terminal innervation is present in the intermediate reticular nucleus (IRN), parvicellular reticular nucleus (PARN), and medullary reticular nucleus dorsal part (MDRNd), and lighter inputs reach the facial nucleus (VII), magnocellular reticular nucleus (MARN), gigantocellular reticular nucleus (GRN), medial vestibular nucleus (MV), and spinal nucleus of trigeminal nerve oral part (SPVO). Axons from MOp-ul not terminating in these regions continue through the pyramidal decussation to densely innervate the contralateral reticular formation generally and the cuneate nucleus specifically. The remaining MOp-ul axons then contribute to the spinal portion of the corticospinal tract, continuing caudally to reach the intermediate spinal zone and ventral horn of the cervical spinal cord, where they terminate (**Extended Data Fig. 5l**).

Stereotaxic surgeries, histology and immunohistochemical processing (Allen Institute)

Mice were anesthetized with 5% isoflurane and placed into a stereotaxic frame (Model# 1900, Kopf, Tujunga, CA). The isoflurane level was maintained at 1-5% throughout the surgery. For anterograde viral tracer experiments and for helper virus delivery for retrograde trans-synaptic tracing, rAAV was delivered through glass pipettes (inner tip diameters of 10–20 μm) by iontophoresis with current settings of 3 or 5 μA using 7 sec on/off cycles for 5 min total. For retrograde trans-synaptic tracing, rabies virus was delivered by pressure injection (Nanoject II Variable Volume Automatic Injector, VWR) in 23 nl increments over a 3 min and 10 sec interval to a final volume of 500 nl. All injections were placed in the right hemisphere. MOp-ul was targeted using stereotaxic coordinates: anterior/posterior 0.62 mm from Bregma, medial/lateral 1.5 mm from the midline at Bregma, and dorsal/ventral (DV) 0.3 or 0.6 mm from the pial surface of the brain.

Anterograde tracing brains were collected 3 weeks from the AAV injection date. Retrograde trans-synaptic input mapping brains were collected 9 days from the rabies virus injection date. All mice were deeply anesthetized with 5% isoflurane and intracardially perfused with 10 ml of 0.9% saline solution followed by 50 ml of 4% PFA at a flow rate of 9 ml/min. Brains were rapidly dissected and post-fixed in 4% PFA at room temperature for 3-6 hours and overnight at 4°C. Brains were then rinsed briefly with PBS and stored in PBS with 0.02% sodium azide until tissue preparation for serial two photon imaging (see Imaging and post-acquisition processing in Methods).

For rabies starter cell imaging and counting, sections were collected following serial two-photon imaging, mounted on gelatin coated glass slides and processed for immunofluorescence using an automated slide stainer (Biocare, IntelliPATH FLX). Slides were placed in a blocking solution of Image iT FX Signal Enhancer (Thermo Fisher Scientific Cat# I3693) for 45 minutes, followed by a blocking solution containing 1% normal goat serum (Vector Laboratories Cat#S1000) and 1% Triton X (VWR) for 1 hour. Sections were then incubated in dsRed primary antibody solution (1% goat serum, 1% Triton X, 1:2000 rabbit anti-dsRed antibody (Rockland Cat# 600-401-379, RRID:AB_2209751) for 1.5 hours at room temperature. Slides were rinsed in 0.1% Triton X wash solution and then incubated in secondary antibody solution (1% goat serum, 1% Triton X, and 1:500 goat anti-rabbit conjugated with Alexa Fluor 594 (Thermo Fisher Scientific Cat# A-11037, RRID:AB_2534095) for 2 hours. Finally, all sections were stained with 5 μM Dapi (Thermo Fisher Scientific D1306) and coverslipped using Fluoromount G (Southern Biotech Cat# 0100-01B).

Stereotaxic surgeries, histology and immunohistochemical processing (Huang Lab at CSHL)

Adult mice were anesthetized by inhalation of 2% isofluorane delivered with a constant air flow (0.4 L•min-1). Ketoprofen (5 mg•kg-1) and dexamethasone (0.5 mg•kg-1) were administered subcutaneously as preemptive analgesia and to prevent brain edema, respectively, prior to surgery, and lidocaine (2-4 mg•kg-1) was applied intra-incisionally. Mice were mounted in a stereotaxic headframe (Kopf Instruments, 940 series or Leica Biosystems, Angle Two). Stereotactic coordinates of MOp-ul were identified. An incision was made over the scalp, a small burr hole drilled in the skull and brain surface exposed.

A pulled glass pipette tip of 20–30 μm containing the viral suspension was lowered into the brain; a 300-400 nl volume was delivered at a rate of 30 nl•min-1 using a Picospritzer (General Valve Corp); the pipette remained in place for 10 min preventing backflow, prior to retraction, after which the incision was closed with 5/0 nylon suture thread (Ethilon Nylon Suture, Ethicon Inc. Germany) or Tissueglue (3M Vetbond), and animals were kept warm on a heating pad until complete recovery. Details on the virus used per animal can be found in **Supplementary Tables 2 and 3**.

For cell-type specific mono-trans-synaptic rabies tracing of inputs, postnatal mice aged 2 months were anesthetized using Avertin and intracardially perfused with saline followed by 4% PFA in PBS; brains were post-fixed in 4% PFA overnight at 4 ºC and subsequently rinsed three times, embedded in 3% agarose-PBS and cut 50–100 μm in thickness using a vibrating microtome (Leica, VT100S). Sections were placed in blocking solution containing 10% Normal Goat Serum (NGS) and 0.1% Triton-X100 in PBS1X for 1 hr, then incubated overnight at 4 ºC with primary antibodies diluted blocking solution. Anti-GFP (1:1000, Aves, GFP-1020); anti-RFP (1:1000, Rockland Pharmaceuticals, 600-401-379) were used. Sections were rinsed 3 times in PBS and incubated for 1 hr at room temperature with corresponding secondary antibodies (1:500, Life Technologies). Sections were washed three times with PBS and incubated with DAPI for 5 min (1:5,000 in PBS, Life Technologies, 33342) to stain nuclei. Sections were dry-mounted on slides using Vectashield (Vector Labs, H1000) or Fluoromount (Sigma, F4680) mounting medium.

Supplementary Methods and data analysis of BARseq (Zador lab at CSHL)

Supplementary methods

Male mice at age of 56 days were utilized for all experiments. Two mice were injected in the MOp using coordinate: anterior 0.5 mm from the Bregma, 1.5 mm mediolateral from the midline, and depth at 500 μm and 900 μm. At each depth, 150 nL of a JK100L2 Sindbis barcoded library[16,17] diluted 1:2 in PBS was injected. After 24 hrs, each animal was sacrificed, and a 3 mm diameter biopsy punch was used to remove the area around the injection site. The injection site punch was mounted in OCT and snap-frozen in liquid nitrogen – isopentane bath. The rest of the brain was frozen on dry ice, cryo-sectioned to 300 μm slices, and processed for projection site sequencing.

The projection sites were dissected manually based on a list of targets designed to match bulk GFP tracing results. Several large brain areas, including the striatum, the thalamus, and the medulla, were dissected into multiple samples to obtain a relative position of the projections. A list of designed dissection sites and their coordinates is provided in **Supplementary Table 6**. The images of the slices after dissection are provided at Mendeley data (see data presentation). After dissection, RNA barcodes were extracted from the samples, amplified, and sequenced as described previously[16]. The raw sequencing data and processed barcode counts are available from Sequence Read Archive (SRA) and Mendeley data (see data presentation).

The injection site was cryo-sectioned to 20 μm sections and the sections were mounted onto Superfrost Plus Gold slides (Electron Microscopy Sciences) using NOA81 glue and a home-made tape-transfer system[18]. Six slides encompassing the center of the injection site were then processed for BARseq library preparation and sequencing as previously described[16].

Sequencing images were captured on an Olympus IX81 microscope with a Crest X-light v2 spinning disk confocal, an 89north LDI 7-channel laser, and a Photometrics Prime BSI camera. Image acquisition was controlled through micro-manage[19]. Filters and lasers used for each imaging channel are provided in **Supplementary Table 7**. At each sequencing cycle, a z-stack with a step size of 7 μm and a span of 50 μm centered on the injection site was taken using a UPLSAPO 10× NA 0.45 objective for all four sequencing channels and the DIC channel. After the last sequencing cycle is done, we performed DAPI staining and imaged a 3 × 3 tile with 15% overlap to roughly cover the whole punch.

To process the images, we first max-projected all z-stacks, followed by median filtering, channel bleed-through correction, and top-hat filtering. The pre-processed images, including the stitched image of the 3 ×3 tile, were then registered to the first sequencing cycle using ECC image alignment[20]. Cells were identified by first finding local maxima above a prominence threshold, then segmenting somata using the sequencing signals around each local maxima. The barcodes were base-called by calling the channel with the maximum signal in each sequencing cycle across all pixels within the segmented cells. We then manually marked the outline of the top and the bottom of the cortex, and the medial and lateral boundaries of the punch in the stitched 3 × 3 images. The distance of each cell to these four boundaries were determined to find the relative position of the cell within the cortex. The *in situ* barcodes were matched to the barcodes at the projection sites, allowing one mismatch but no ambiguous matches (i.e. an *in situ* barcode cannot match to two projection barcodes with equal hamming distance). Only *in situ* barcodes with a minimum quality score of at least 0.8 across all cycles were kept for further analysis.

Some barcodes were seen in more than one cell. This could be caused by a cell being sliced into two in adjacent slices, or the apical dendrite of a cell being segmented as a cell body by mistake, or by two cells being labeled with the same barcode. The latter two possibilities would either cause the cell to be identified in the wrong laminar position or a loss of single-cell resolution in projection mapping. We thus removed all pairs of cells with the same barcodes if they had a laminar difference of more than 100 μm or were not found on the same or adjacent slices. For the remaining pairs, we kept the cell in the deeper layer (because apical dendrites were likely more superficial than the somata). We further filtered the cells so that all cells have a strongest projection of at least 5 molecule counts. This resulted in 10,299 projection neurons for further analyses.

Analysis of BARseq compared to single-cell tracing and retrograde labeling

Consistent with retrograde labeling, BARseq revealed that MOp projections were enriched in different sublayers (**Extended Data Fig. 18a**). To compare projections across the 11 sublayers defined by retrograde tracing, we first defined

each layer boundary according to differences in projections to a single target based on retrograde labeling. We then examined whether all projections, including the layer-defining projections, were differentially enriched across neighboring sublayers. For each pair of adjacent sublayers (except L1 and L2, because L1 has no projection neurons), we found at least three projection targets that were differentially innervated by neurons in the two adjacent sublayers (**Extended Data Fig. 18b**). These targets included those that were not used in defining the sublayer boundaries. For example, the boundary between L5a and L5b-s was defined using projections to the superior colliculus (SC), but L5b-s also had stronger projections to the striatum, thalamus, various midbrain targets, the medulla, and the spinal cord. These results further confirmed that projections were distinct across the 11 sublayers.

To assess whether the projection patterns obtained by BARseq were comparable to those obtained by single-cell pathway tracing, we randomly downsampled the BARseq dataset almost 100-fold to obtain the same sample size as a subset of the single-cell tracing data (about 160 neurons), and then analyzed the combined dataset at a common axonal resolution achieved by both datasets (**Extended Data Fig. 18c, d**; see **Methods**). BARseq neurons were intermingled with traced neurons in t-SNE space (**Extended Data Fig. 18e, f**). Furthermore, clustering the combined dataset produced clusters that contained neurons of both datasets at both the major class-level divisions and at the subtype level (**Extended Data Fig. 18g**). These analyses indicate that the single-neuron projection patterns obtained by BARseq were consistent with those obtained by single-neuron pathway tracing.

BARseq, in its current form, has much higher throughput than current single-cell pathway tracing techniques, but at the expense of axonal resolution. However, despite the reduced axonal resolution, BARseq identified 18 groups of neurons with differential laminar distributions (**Fig. 4b, c**), compared to six clusters identified by this subset of single-cell pathway tracing—to a certain level of resolution. The 18 identified groups largely matched the high-level divisions of tracing data obtained using MetaNeighbor [21] (**Extended Data Fig. 18h**). To confirm that the additional groups identified in BARseq indeed reflect structures in projections not captured by clustering the single-cell tracing data, we examined how downsampled data captured information in the full clusters. Specifically, we focused on two BARseq groups (group 14 and group 7), both of which matched well to cluster 5 of the single-cell tracing data using MetaNeighbor (**Extended Data Fig. 18i**). When downsampling both datasets to the same number of neurons, BARseq subsamples better recapitulated the full cluster information compared to tracing subsamples (**Extended Data Fig. 18j).** Furthermore, the similarities of clusters across the two datasets were also preserved better in subsamples of BARseq neurons compared to subsamples of tracing neurons with the same number of cells (**Extended Data Fig. 18k**). These results suggest that projection groups in BARseq were more homogeneous than the tracing clusters and reflected additional structures in projection patterns. The ability of BARseq to capture additional structures in projections by sampling projection patterns more broadly, but less deeply, is reminiscent of similar observations among single-cell RNAseq techniques[22,23].

Most (81%) IT neurons projected to two or more of the 39 sampled target areas (**Extended Data Fig. 19a**) with some neurons (1.9% of neurons) projecting to 11 or more areas. The most common binary projection patterns were dominated by combinations of projections to ipsilateral MOs, SSs, and contralateral MOp, consisting of seven out of the top eight binary projection patterns (**Extended Data Fig. 19b**). These top eight binary patterns included 34% of all IT neurons (**Extended Data Fig. 19c**). Because our analysis would miss projections to areas that BARseq did not sample, our estimates of number projection targets per neuron represent a lower bound.

For L5 ET neurons, we found two types (groups 16 and 18) with distinct laminar positions (**Extended Data Fig. 19e**). Group 16 neurons were mostly located in superficial L5b and projected to the thalamus. In addition, this group also contains the majority of neurons projecting to the pons but not to the medulla (30 out of 123 neurons in group 16, compared to 8 out of 766 neurons in group 18, $p = 1.1\times10^{-20}$ using Fisher's test). Group 18 neurons were in middle and deep L5b and projected predominantly to the medulla. Furthermore, heterogeneity in the projections of group 18 neurons predicted their sublaminar positions: Neurons with stronger projections to the midbrain reticular nucleus (MRN), the striatum, the thalamus, and zona incerta (ZI) were located more superficially whereas neurons with stronger projections to the medulla and the spinal cord were located in the L5b-deep sublayer (**Extended Data Fig. 19f**). These differences in laminar distribution were unlikely to be an artefact caused be potential contamination by group 16, because the MRN and the striatum were more likely to be co-innervated with the medulla and pons than with the thalamus and ZI (**Extended Data Fig. 19g**). These results are thus consistent with retrograde tracing results and single neuron morphology data and further revealed sublaminar organization of heterogeneity in L5 ET projections beyond the two types identified by other studies and data modalities[24-27].

The higher order structure in the projections of IT neurons is highly informative of the laminar locations of neurons. For example, IT neurons that project to the striatum bilaterally have overlapping but differential distribution across L5 compared with those projecting to the ipsilateral striatum only (**Extended Data Fig. 19h**). In a second example, the laminar positions of IT neurons were strongly dependent on projections to the secondary somatosensory cortex, but the relationship between such projections and the laminar positions of neurons were reversed in neurons with or without contralateral projections. Neurons projecting only to the ipsilateral cortex, but not the secondary somatosensory cortex

(group 14), were confined to a thin layer at the bottom of L6 (**Extended Data Fig.19i**) (this is also consistent with retrograde tracing results as shown in **Fig. 1d & Extended Data Fig. 3b,c**, whereas those projecting to the ipsilateral secondary somatosensory cortex (groups 13 and 15) were mostly in L2/3. Conversely, callosal neurons projecting to the somatosensory cortex were more enriched in L6 compared to those not projecting to the secondary somatosensory cortex (**Extended Data Fig.19j**). We did not observe consistent bias in the distribution of subgroups in the tangential plane (**Extended Data Fig. 19k**). The overall projection patterns of IT neurons thus reveal additional layer specificity not predicted by the distribution of projections to individual areas alone.

Supplementary Table 6. List of dissected projection targets in BARseq experiments and their corresponding section numbers in the Allen Reference Atlas (ARA). Abbreviations can be found in Supplementary Table 1.

	ARA area labels	Area name	ARA slice numbers	Annotations
1	/	OB	/	Negative control, one slice.
Cortex				
2	ORBvl/ORBl/ORBm	ORB-ipsi	31-37	
3	ORBvl/ORBl/ORBm	ORB-contra	31-37	
4	MOs	MOs-rostral-ipsi	23-35	
5	MOs	MOs-rostral-contra	23-35	
6	CLA	CLA-ipsi	40-46	Could not separate confidently from CP.
7	CLA	CLA-contra	40-46	
8	MOp	MOp-contra	44-59	
9	SSs	SS-ipsi	58-70	
10	SSs	SS-contra	58-70	
11	SSp	SSp-ipsi	60-69	Unable to collect at the intended site (the area was removed by injection site punch); excluded from analysis.
12	SSp	SSp-contra	60-69	
13	Tea/ECT/PERI/ENTl	TEa-ipsi	76-95	
14	Tea/ECT/PERI/ENTl	TEa-contra	76-95	
Striatum				
15	CP	Str-r-ipsi	39-47	The R/I/C boundaries are chosen to be in the middle of R/I/C slices in Hintiryan et al 2016 to maximize compatibility.
16	CP	Str-r-contra	39-47	
17	CP	Str-i-ipsi	48-56	
18	CP	Str-i-contra	48-56	
19	CP	Str-c-ipsi	57-72	
20	CP	Str-c-contra	57-72	
Thalamus				
21	TH	Thal-mr-ipsi	63-69	Rostral Thalamus separated into medial and lateral divisions.
22	TH	Thal-lr-ipsi	63-69	

23	TH	Thal-mr-contra	63-69	
24	TH	Thal-lr-contra	63-69	
25	TH	Thal-mc-ipsi	73-80	
26	TH	Thal-lc-ipsi	73-80	
27	TH	Thal-mc-contra	73-80	
28	TH	Thal-lc-contra	73-80	
29	ZI	ZI-ipsi	80-83	
Midbrain				
30	MRN/RN/PAG/SC	MRN	83-89	Includes most of midbrain and lateral side of SC, can increase dissected volume within midbrain only.
31	SC	SC	96-103	Top of SC. Note that this is more posterior to MRN to increase separation of the sites.
32	PG	PG	90-97	
Medulla				
33	MY	MY-l-ipsi	108-132	Corticospinal tract excluded (i.e. some minor exclusion of MY close to the tract) and MY separated into medial/lateral parts on either side.
34	MY	MY-m-ipsi	108-132	
35	MY	MY-l-contra	108-132	
36	MY	MY-m-contra	108-132	
Spinal cord				
37	/	Sp-cervical	/	
28	/	Sp-thoracic	/	
29	/	Sp-lumbar	/	
40	/	Sp-sacral	/	

Supplementary Table 7. Lasers and filters used for imaging.

Channel	Laser (nm)	Excitation filter	Dichroic filter	Emission filter
DAPI	405	zet402/468/555/640x (Chroma)	Zt402/468/555/640rpc-ufs (Chroma)	Zet402/648/555/640m (Chroma)
G	520	Zet443-518x (Chroma)	Zt443-518rpc (Chroma)	FF01-565/24 (Semrock)
T	555	zet402/468/555/640x (Chroma)	Zt402/468/555/640rpc-ufs (Chroma)	FF01-585/11 (Semrock)
A	640	zet402/468/555/640x (Chroma)	FF652-Di01 (Semrock)	FF01-676/29 (Semrock)
C	640	zet402/468/555/640x (Chroma)	FF652-Di01 (Semrock)	FF01-725/40

Supplementary Table 8. P-values for Extended Data Fig. 18b

	OB	MOsrostral-ipsi	ORB-ipsi	CLA-ipsi	SS-ipsi	TEa-ipsi	MOsrostral-contra	ORB-contra
L1/L2	NaN	5.93E-01	1.00E+00	NaN	4.62E-02	1.00E+00	8.94E-01	9.50E-01
L2/L3	NaN	1.61E-08	1.18E-01	5.05E-01	8.88E-05	1.18E-01	3.47E-01	3.99E-01
L3/L4	NaN	3.95E-04	1.24E-02	1.62E-01	7.92E-02	5.02E-02	3.58E-01	6.90E-02
L4/L5a	NaN	4.19E-11	6.73E-02	2.85E-11	9.16E-04	3.20E-11	5.97E-11	6.88E-02
L5a/L5b-s	1.53E-01	4.87E-12	7.09E-01	1.86E-24	8.74E-12	2.35E-04	2.88E-03	7.27E-05
L5b-s/L5b-m	5.11E-01	2.22E-10	1.06E-01	1.84E-05	5.49E-09	6.26E-05	9.39E-04	1.35E-01
L5b-m/L5b-d	NaN	8.86E-02	7.31E-01	3.96E-02	1.13E-01	5.98E-02	3.18E-02	3.41E-01
L5b-d/L6a-s	NaN	1.32E-23	7.64E-02	3.05E-17	3.68E-15	2.82E-01	2.02E-08	4.51E-08
L6a-s/L6a-d	NaN	3.58E-01	7.85E-01	4.85E-01	3.62E-04	8.63E-08	5.53E-04	5.86E-02
L6a-d/L6b	NaN	9.72E-52	3.49E-03	9.68E-01	7.57E-02	2.42E-01	4.20E-01	1.61E-01
	CLA-contra	MOp-contra	SS-contra	SSp-contra	TEa-contra	Str-r-ipsi	Str-i-ipsi	Str-c-ipsi
L1/L2	NaN	3.53E-01	9.50E-01	NaN	8.73E-01	1.00E+00	8.09E-01	7.49E-01
L2/L3	7.02E-01	1.30E-19	5.93E-01	5.05E-01	3.64E-06	6.41E-01	7.00E-01	4.84E-01
L3/L4	3.79E-01	2.56E-09	3.28E-01	7.55E-01	2.56E-01	2.82E-04	9.83E-22	1.54E-20
L4/L5a	9.80E-13	1.30E-02	5.00E-12	1.14E-01	1.42E-09	8.69E-34	8.12E-97	1.50E-148
L5a/L5b-s	3.27E-37	1.50E-01	9.32E-03	7.77E-01	1.19E-03	1.84E-02	4.90E-35	1.80E-38
L5b-s/L5b-m	8.62E-02	3.29E-02	3.97E-03	6.26E-01	9.71E-03	1.61E-10	1.88E-22	1.85E-34
L5b-m/L5b-d	7.57E-03	9.31E-01	9.96E-01	6.64E-01	2.79E-02	4.60E-02	2.68E-12	1.01E-07
L5b-d/L6a-s	7.01E-29	6.19E-47	3.76E-08	4.72E-01	8.04E-01	2.82E-24	3.12E-75	9.18E-29
L6a-s/L6a-d	3.48E-05	1.09E-12	6.50E-04	1.91E-01	1.06E-01	5.90E-02	2.10E-01	1.59E-03
L6a-d/L6b	4.20E-01	2.07E-09	1.77E-01	1.61E-01	2.84E-01	3.70E-01	4.09E-02	6.79E-03
	Str-r-contra	Str-i-contra	Str-c-contra	Thal-mr-ipsi	Thal-lr-ipsi	Thal-mc-ipsi	Thal-lc-ipsi	Thal-mr-contra
L1/L2	NaN	1.00E+00	1.00E+00	NaN	1.00E+00	1.00E+00	1.00E+00	NaN
L2/L3	5.87E-01	1.18E-01	9.56E-03	7.02E-01	1.18E-01	9.56E-03	9.56E-03	NaN
L3/L4	2.55E-01	7.72E-04	2.31E-02	8.58E-01	4.23E-01	2.57E-01	2.57E-01	NaN
L4/L5a	1.26E-18	7.76E-26	2.41E-15	1.83E-02	2.87E-04	4.67E-03	1.91E-03	6.04E-01
L5a/L5b-s	1.71E-32	5.96E-71	4.21E-12	5.45E-11	1.11E-38	4.00E-32	5.32E-27	2.90E-03
L5b-s/L5b-m	4.28E-03	8.19E-04	1.16E-07	2.42E-02	1.07E-02	3.31E-01	1.45E-01	8.38E-01
L5b-m/L5b-d	2.53E-04	3.17E-06	1.51E-01	5.70E-02	2.48E-05	2.45E-02	4.33E-06	6.18E-01
L5b-d/L6a-s	8.71E-34	1.51E-59	1.78E-15	4.12E-30	9.32E-111	2.00E-60	3.06E-107	1.63E-04

L6a-s/L6a-d	9.94E-05	6.44E-07	2.11E-02	1.21E-33	6.42E-01	1.20E-41	1.46E-05	1.06E-16	
L6a-d/L6b	3.36E-01	3.47E-01	4.20E-01	3.07E-02	4.95E-13	5.66E-06	5.05E-12	2.24E-02	
	Thal-lr-contra	Thal-mc-contra	Thal-lc-contra	ZI-ipsi	MRN	PG	SC	MY-l-ipsi	
L1/L2	NaN	NaN	NaN	NaN	NaN	NaN	NaN	NaN	
L2/L3	NaN	NaN	NaN	NaN	NaN	NaN	NaN	NaN	
L3/L4	NaN	NaN	NaN	NaN	NaN	NaN	NaN	NaN	
L4/L5a	NaN	NaN	NaN	1.24E-02	1.83E-04	1.99E-03	4.36E-02	2.45E-01	
L5a/L5b-s	NaN	1.33E-02	1.53E-01	2.56E-22	1.14E-46	1.91E-57	8.84E-15	2.52E-08	
L5b-s/L5b-m	1.29E-01	2.54E-01	5.11E-01	9.50E-01	2.43E-02	3.76E-13	1.64E-01	4.66E-04	
L5b-m/L5b-d	4.39E-01	5.54E-01	NaN	5.15E-02	1.19E-02	6.77E-03	8.54E-01	9.65E-01	
L5b-d/L6a-s	5.07E-02	6.37E-01	NaN	8.79E-12	1.78E-38	5.40E-59	7.76E-10	9.41E-09	
L6a-s/L6a-d	2.73E-06	8.56E-13	1.09E-01	3.80E-02	1.56E-03	6.60E-06	1.62E-01	2.12E-03	
L6a-d/L6b	6.42E-02	1.29E-01	1.22E-02	NaN	1.45E-01	2.17E-01	NaN	NaN	
	MY-m-ipsi	MY-l-contra	MY-m-contra	Sp-cervical	Sp-thoracic	Sp-lumbar	Sp-sacral		
L1/L2	NaN	NaN	NaN	NaN	NaN	NaN	NaN		
L2/L3	NaN	NaN	NaN	5.87E-01	NaN	NaN	NaN		
L3/L4	NaN	NaN	NaN	2.13E-01	NaN	NaN	NaN		
L4/L5a	1.69E-02	1.41E-01	1.24E-02	5.13E-02	3.68E-01	NaN	6.04E-01		
L5a/L5b-s	1.57E-51	1.61E-16	6.10E-48	1.50E-36	4.85E-10	1.39E-03	4.84E-01		
L5b-s/L5b-m	7.35E-16	7.74E-14	6.70E-16	1.63E-24	9.86E-14	9.86E-05	1.29E-01		
L5b-m/L5b-d	1.08E-02	4.80E-01	2.00E-02	2.74E-02	1.90E-01	8.76E-01	9.18E-02		
L5b-d/L6a-s	4.56E-55	4.45E-29	5.02E-55	7.41E-55	3.20E-23	2.30E-07	NaN		
L6a-s/L6a-d	6.60E-06	1.05E-04	6.60E-06	8.17E-06	1.22E-02	1.62E-01	NaN		
L6a-d/L6b	NaN	2.17E-01	2.17E-01	2.17E-01	NaN	NaN	NaN		

References for Supplementary Information.

1 Tward, D. *et al.* Diffeomorphic registration with intensity transformation and missing data: Application to 3D digital pathology of Alzheimer's disease. *Frontiers in neuroscience* **14**, 52 (2020).

2 Lein, E. S. *et al.* Genome-wide atlas of gene expression in the adult mouse brain. *Nature* **445**, 168-176 (2007).

3 Kuan, L. *et al.* Neuroinformatics of the Allen Mouse Brain Connectivity Atlas. *Methods* 73, 4-17 (2015).

4 Wang, Q. *et al.* The Allen Mouse Brain Common Coordinate Framework: A 3D Reference Atlas. *Cell* (2020).
5 Park, Y.-G. *et al.* Protection of tissue physicochemical properties using polyfunctional crosslinkers. *Nature biotechnology* **37**, 73-83 (2019).
6 Yun, D. H. *et al.* Ultrafast immunostaining of organ-scale tissues for scalable proteomic phenotyping. *bioRxiv*, 660373 (2019).
7 Swaney, J. *et al.* Scalable image processing techniques for quantitative analysis of volumetric biological images from light-sheet microscopy. *BioRxiv*, 576595 (2019).
8 Kim, Y. *et al.* Mapping social behavior-induced brain activation at cellular resolution in the mouse. *Cell Rep* **10**, 292-305, doi:10.1016/j.celrep.2014.12.014 (2015).
9 Dong, H. W. *The Allen reference atlas: A digital color brain atlas of the C57Bl/6J male mouse.* (John Wiley & Sons Inc, 2008).
10 Chon, U., Vanselow, D. J., Cheng, K. C. & Kim, Y. Enhanced and unified anatomical labeling for a common mouse brain atlas. *Nature communications* **10**, 1-12 (2019).
11 Morandell, K. & Huber, D. The role of forelimb motor cortex areas in goal directed action in mice. *Scientific reports* **7**, 1-14 (2017).
12 Hintiryan, H. *et al.* The mouse cortico-striatal projectome. *Nature neuroscience* **19**, 1100-1114 (2016).
13 Hoerder-Suabedissen, A. *et al.* Subset of cortical layer 6b neurons selectively innervates higher order thalamic nuclei in mice. *Cerebral Cortex* **28**, 1882-1897 (2018).
14 Reep, R., Corwin, J., Hashimoto, A. & Watson, R. Efferent connections of the rostral portion of medial agranular cortex in rats. *Brain research bulletin* **19**, 203-221 (1987).
15 Alloway, K. D., Olson, M. L. & Smith, J. B. Contralateral corticothalamic projections from MI whisker cortex: potential route for modulating hemispheric interactions. *Journal of Comparative Neurology* **510**, 100-116 (2008).
16 Chen, X. *et al.* High-throughput mapping of long-range neuronal projection using in situ sequencing. *Cell* **179**, 772-786. e719 (2019).
17 Kebschull, J. M. *et al.* High-Throughput Mapping of Single-Neuron Projections by Sequencing of Barcoded RNA. *Neuron* **91**, 975-987, doi:10.1016/j.neuron.2016.07.036 (2016).
18 Pinskiy, V. *et al.* High-throughput method of whole-brain sectioning, using the tape-transfer technique. *PloS one* **10**, e0102363 (2015).
19 Edelstein, A. D. *et al.* Advanced methods of microscope control using μManager software. *Journal of biological methods* **1** (2014).
20 Evangelidis, G. D. & Psarakis, E. Z. Parametric image alignment using enhanced correlation coefficient maximization. *IEEE Transactions on Pattern Analysis and Machine Intelligence* **30**, 1858-1865 (2008).
21 Crow, M., Paul, A., Ballouz, S., Huang, Z. J. & Gillis, J. Characterizing the replicability of cell types defined by single cell RNA-sequencing data using MetaNeighbor. *Nature communications* **9**, 1-12 (2018).
22 Yao, Z. *et al.* An integrated transcriptomic and epigenomic atlas of mouse primary motor cortex cell types. *bioRxiv*, 2020.2002.2029.970558, doi:10.1101/2020.02.29.970558 (2020).
23 Ding, J. *et al.* Systematic comparison of single-cell and single-nucleus RNA-sequencing methods. *Nature biotechnology*, 1-10 (2020).
24 Economo, M. N. *et al.* A platform for brain-wide imaging and reconstruction of individual neurons. *Elife* **5**, e10566, doi:10.7554/eLife.10566 (2016).
25 Tasic, B. *et al.* Shared and distinct transcriptomic cell types across neocortical areas. *Nature* **563**, 72-78 (2018).
26 Zhang, M. *et al.* Molecular, spatial and projection diversity of neurons in primary motor cortex revealed by in situ single-cell transcriptomics. *bioRxiv*, 2020.2006.2004.105700, doi:10.1101/2020.06.04.105700 (2020).
27 Adkins, R. M., Gelke, E. L., Rowe, D. & Honeycutt, R. L. Molecular phylogeny and divergence time estimates for major rodent groups: evidence from multiple genes. *Molecular Biology and Evolution* **18**, 777-791 (2001).
