# Supplementary material for: Cellular anatomy of the mouse primary motor cortex
Source: Nature. 2021 Oct 6;598(7879):159–66. doi: 10.1038/s41586-021-03970-w (PMC8494646; doi:10.1038/s41586-021-03970-w)
Supplement: Supplementary file 2 — Reporting Summary [file 41586_2021_3970_MOESM2_ESM.pdf]

nature portfolio

Corresponding author(s): Hong-Wei Dong, Julie Harris, Pavel Osten, Josh Huang, Giorgio Ascoli

Last updated by author(s): 08/24/2021

Reporting Summary

Nature Portfolio wishes to improve the reproducibility of the work that we publish. This form provides structure for consistency and transparency in reporting. For further information on Nature Portfolio policies, see our Editorial Policies and the Editorial Policy Checklist.

Statistics

For all statistical analyses, confirm that the following items are present in the figure legend, table legend, main text, or Methods section.

n/a	Confirmed	
☐	☒	The exact sample size (*n*) for each experimental group/condition, given as a discrete number and unit of measurement
☐	☒	A statement on whether measurements were taken from distinct samples or whether the same sample was measured repeatedly
☐	☒	The statistical test(s) used AND whether they are one- or two-sided<br>*Only common tests should be described solely by name; describe more complex techniques in the Methods section.*
☒	☐	A description of all covariates tested
☐	☒	A description of any assumptions or corrections, such as tests of normality and adjustment for multiple comparisons
☐	☒	A full description of the statistical parameters including central tendency (e.g. means) or other basic estimates (e.g. regression coefficient) AND variation (e.g. standard deviation) or associated estimates of uncertainty (e.g. confidence intervals)
☐	☒	For null hypothesis testing, the test statistic (e.g. *F*, *t*, *r*) with confidence intervals, effect sizes, degrees of freedom and *P* value noted<br>*Give P values as exact values whenever suitable.*
☒	☐	For Bayesian analysis, information on the choice of priors and Markov chain Monte Carlo settings
☒	☐	For hierarchical and complex designs, identification of the appropriate level for tests and full reporting of outcomes
☐	☒	Estimates of effect sizes (e.g. Cohen's *d*, Pearson's *r*), indicating how they were calculated

Our web collection on statistics for biologists contains articles on many of the points above.

Software and code

Policy information about availability of computer code

Data collection	Several microscopic methods were used to collect fluorescent imaging data: 1). epifluorescence images were collected with the Olympus VS120 fluorescence microscope running Olympus VS-Desktop v2.9; 2)High resolution confocal images were captured using an Andor DragonFly 202 spinning disk confocal microscope running Fusion v2.1.0.81 software; 3) Lightsheet images were captured with a LifeCanvas lightsheet microscope running SmartSPIM Acquisition Software 2019V3 and oblique Light-sheet tomography (OLST) running custom open source software; 4) 3D fluorescent-labeled pathway images were collected using Serial Two-Photon Tomography (STPT) instruments with TissueVision software 5) single neuron morphology data were collected using fluorescence micro-optical sectioning tomography system (fMOST); 6)BARseq data were collected using an Olympus IX81 microscope with a Crest X-light v2 spinning disk confocal, an 89north LDI 7-channel laser, and a Photometrics Prime BSI camera. Image acquisition was controlled through micro-manager.<br>STPT images at the Allen Institute were processed using the Allen informatics data pipeline (IDP), which manages the processing and organization of the images and quantified data for analysis and display in the web application as previously described (Oh et al., 2014 and Kuan et al. 2015). STPT images at CSH were porcessed with custom open source OpenSTPT software.
Data analysis	We used various computational/informatics methods for data analysis, which are all described in detail in Methods section. Novel code will be made publicly accessible through Github or other repositories as indicated in the Methods.<br>For Figure 4, 5, and 7, unsupervised hierarchical clustering was conducted with the online software, Morpheus, (https://software.broadinstitute.org/morpheus/) for algorithms and for visualization of the dendrogram. The software program GraphPad Prism was used for statistical tests and generation of graphs.

For manuscripts utilizing custom algorithms or software that are central to the research but not yet described in published literature, software must be made available to editors and reviewers. We strongly encourage code deposition in a community repository (e.g. GitHub). See the Nature Portfolio guidelines for submitting code & software for further information.

Data

Policy information about availability of data

All manuscripts must include a data availability statement. This statement should provide the following information, where applicable:

- Accession codes, unique identifiers, or web links for publicly available datasets
- A description of any restrictions on data availability
- For clinical datasets or third party data, please ensure that the statement adheres to our policy

All the data underlying the results described in this work will be or already are deposited in the Brain Image Library at the Pittsburgh Supercomputing Center and publicly accessible without restrictions or credentials at biccn.org/data through the BICCN Data Inventory hosted by the Brain Cell Data Center at the Allen Institute for Brain Science.

Field-specific reporting

Please select the one below that is the best fit for your research. If you are not sure, read the appropriate sections before making your selection.

☒ Life sciences ☐ Behavioural & social sciences ☐ Ecological, evolutionary & environmental sciences

For a reference copy of the document with all sections, see nature.com/documents/nr-reporting-summary-flat.pdf

Life sciences study design

All studies must disclose on these points even when the disclosure is negative.

Sample size	The sample sizes for different injection methods with different tracers were specified in Methods sections as described for different laboratories.
Data exclusions	The best most representative injections were chosen for the analysis. The others were excluded due to off-targeting of the injection site, missing/damaged tissue, weak tracer labeling of the axons or high background, etc.
Replication	This study focuses on characterizing inputs/outputs of the primary motor cortex upper limb area (MOp-ul) using different tracing methods. Each of tracer injections were repeated multiple times in different animals. While the best, most representative cases were chosen for inclusion in the analysis data set, the other injections served as validation cases, demonstrating the replicability and consistency of tracer labeling.
Randomization	Randomization is not relevant to the present work since animals were not compared across different conditions
Blinding	Traditional blinding was not necessary since animals were not compared across different conditions in this study.

Reporting for specific materials, systems and methods

We require information from authors about some types of materials, experimental systems and methods used in many studies. Here, indicate whether each material, system or method listed is relevant to your study. If you are not sure if a list item applies to your research, read the appropriate section before selecting a response.

Materials & experimental systems

n/a	Involved in the study	
☐	☒	Antibodies
☒	☐	Eukaryotic cell lines
☒	☐	Palaeontology and archaeology
☐	☒	Animals and other organisms
☒	☐	Human research participants
☒	☐	Clinical data
☒	☐	Dual use research of concern

Methods

n/a	Involved in the study	
☒	☐	ChIP-seq
☒	☐	Flow cytometry
☒	☐	MRI-based neuroimaging

Antibodies

Antibodies used	[antibody; vendor; catalog number]<br>1. rabbit anti-Phaseolus vulgaris leucoagglutinin antibody; Vector Labs; #AS-2300<br>2. monoclonal mouse anti-Cre recombinase, clone 2D8; Millipore Sigma; #MAB3120<br>3. rabbit anti-dsRed antibody, Rockland Cat# 600-401-379, RRID:AB_2209751<br>4. Mouse anti-Neurofilament-M antibody (monoclonal), Encor biotechnology, #MCA-3H11

5. Guinea pig anti-NeuN antibody (Polyclonal), Synaptic systems, #266004

Validation

Supporting documentation as to the validity of the above antibodies can be found at the following:
1. https://antibodyregistry.org/search.php?q=AB_2313686
2. https://antibodyregistry.org/search.php?q=AB_2085748 and https://www.emdmillipore.com/US/en/product/Anti-Cre-Recombinase-Antibody-clone-2D8,MM_NF-MAB3120#documentation
3.https://antibodyregistry.org/search.php?q=AB_2209751
4. https://encorbio.com/product/mca-3h11/
5. https://sysy.com/product/266004

Animals and other organisms

Policy information about studies involving animals; ARRIVE guidelines recommended for reporting animal research

Laboratory animals	Mus musculus, male and female, 2-month old, wild type C57Bl6, Cre driver transgenics and reporters, some obtained from Jackson Laboratories. All animal procedures were performed under Institutional Animal Care and Use Committee (IACUC) approval (Allen Institute for Brain Science, Cold Spring Harbor Laboratory, University of Southern California, MIT, Huazhong University of Science and Technology in China) in accordance with NIH guidelines. Mice had ad libitum access to food and water and were group-housed within a temperature- (21-22°C), humidity- (40-51%), and light- (12hr light/dark cycle) controlled room within the vivariums of the institutes listed above. Male and female wild type C57BL/6J mice at an average age of P56 were purchased from Jackson Laboratories for histological, multi-fluorescent tract tracing and viral tracing experiments, and single neuron reconstructions. The mouse lines used at different institutes for specific experiments are described below and listed in Extended Data Table 2.
Wild animals	No wild animals were used in this study
Field-collected samples	No field samples were collected for this study.
Ethics oversight	Ethical oversight of experimental procedures was performed by the Institutional Animal Care and Use Committee (IACUC) of the Cold Spring Habor Laboratories, University of Southern California, Allen Institute, UCLA, UCSD, MIT, Penn State University, and the Institutional Ethics Committee of Huazhong University of Science and Technology.

Note that full information on the approval of the study protocol must also be provided in the manuscript.
